# The efficacy of Qigong exercises for post-stroke mental disorders and sleep disorders: Protocol for a systematic review and meta-analysis

**DOI:** 10.1097/MD.0000000000021784

**Published:** 2020-08-21

**Authors:** Xing Dong, Renyan Zhang, Ying Guo, Longfang Chen, Yuan Liu

**Affiliations:** a College of Basic Medicine, Chengdu University of Traditional Chinese Medicine, Chengdu, China

**Keywords:** mental disorder, meta-analysis, post-stroke, qigong, sleep disorder, systematic review

## Abstract

**Background:**

Post-stroke mental disorders (PSMDs) and post-stroke sleep disorders (PSSDs) are quite common condition in stroke's patients. Qigong has been widely applied as a replaced and useful treatment for PSMDs and PSSDs. However, the exact effects and safety of Qigong have yet be systematically investigated. Our study focused on summary of efficacy and safety of Qigong for the treatment of advanced PSMDs and PSSDs through the systematic analysis and meta-analysis, in order to provide scientific reference for the clinical.

**Methods:**

The protocol followed Preferred Reporting Items for Systematic Review and Meta-Analyses Protocols. Relevant randomized controlled trials were only considered. Search strategy will be performed in 4 English database including Cochrane Library, PubMed, Web of Science, Excerpt Medical Database, 4 Chinese Database including Chinese Biomedical Literature Database, China National Knowledge Infrastructure, China Scientific Journal Database, Wanfang Database, and WHO International Clinical Trials Registry Platform. Papers in English or Chinese published from their inception to 30 June 2020. Study selection and data extraction will be performed independently by 2 investigators. The clinical outcomes including overall Hamilton depression scale, Hamilton anxiety scale, the mental health part of the MOS item short from health survey, Generic Quality of Life Inventory-74, Center for Epidemiologic Studies Depression Scale, Pittsburgh sleep quality index. Based on the Cochrane Assessment tool and Physiotherapy Evidence Database scale, a modified assessment form should be used to evaluate the methodological quality. Review Manager 5.3 was used for data analysis and risk of bias.

**Results and conclusion:**

We provide some more practical and targeted results examine the effect of Qigong exercises for PSMDs and PSSDs in the relative meta-analysis. We find out defects or inadequacies of Qigong in previous studies. The findings of this research will provide more evidence-based guidance in clinical practice and more rigorous study.

International Platform of Registered Systematic Review and Meta-Analysis Protocols (INPLASY) registration number: INPLASY202070051.

## 1 Introduction

Stroke is the second greatest cause of death following myocardial infarction and 1 of the major causes of disability,^[1]^ and it is reported that this condition will continue until 2030.^[2]^ In the past decades, the number of new-onset stroke people and stroke survivors have been increasing.^[3]^ Because of the large medical burden, current condition of stroke's patients is a significant challenge for governments, especially those in low-income and middle-income countries. China has huge demographic pressure, so the number of stroke patients ranks first in the world. This puts a burden on China's health condition.^[4]^

Survey shows that mental disorders, such as depression, anxiety, phobic disorders, insomnia, and so on are common in stroke patients.^[5–7]^ According to some retrospective literature reported over years, One third of patients after stroke have anxiety and nervousness.^[8,9]^ Comparing with before-stroke, quite a few stroke patients experience less night sleep duration and more daytime sleepiness.^[10]^ Moreover, more and more patients appeared apathy and social inactivity.^[11,12]^ The presence of above mental disorders may bring on not only postponed functional recovery and decline in quality of life, but also higher danger for stroke recrudesce, increased death rate, and cognitive impairment.^[13–16]^ So the enhancing of recognition and management for post-stroke mental disorder (PSMDs) and post-stroke sleep disorder (PSSDs) is absolutely necessary.

Qigong, a theory translated from Chinese,^[17]^ Qigong, as a soft low-impact mind-body aerobic exercise, has been recognized as a “medical” exercise and used to improve physical and psychological health and fight diseases in China for thousands of years.^[18,19]^ There are hundreds of types of Qigong exercises developed in different regions of China. Some are benefit certain diseases while most others have general health benefits. Such as “Muscle/Tendon Changing Classic (Yijinjing),” “The Eight-Section Brocades (Baduanjin),” “The Six Syllable Formula (Liuzijue),” “Five Elements Plam (Wuxinzhang),” “Post Standing Qigong (Zhanzhuang gong),” “Relaxation Qigong (Fangsonggong),” “Internal Nourishing Qigong (Neiyanggong),” “meditation,” “mindfulness,” and “mind concentration”. From the view of western thought and science, this combination of self-awareness with self-correction of the posture and movement of the body be thought to comprise a state that stimulating the balanced release of endogenous neurohormones and a wide range of natural health recovery mechanisms. Actually, many forms of Qigong are using in clinics. Qigong is usually integrated with TCM and with conventional western biomedicine to treat diseases, such as tumor and cancer,^[20,21]^ hypertension,^[22]^ diabetes mellitus,^[23]^ obesity,^[24]^ chronic heart diseases,^[25]^ chronic fatigue syndrome,^[26]^ insomnia,^[27]^ metabolic disease,^[28]^ mental disease,^[29,30]^ and so on. Several complementary medical therapies with some similarities to Qigong are practiced in hospitals in the west and are paid for by insurance.

According to literature reviews, we found that many studies have reported that addition of Qigong could be improve PSMDs and PSSDs patients’ condition. Despite the intensive clinical studies, its clinical efficacy was still not well established and recognized. We are prepared to summarize the efficacy and adverse events of Qigong treatment of PSMDs at advanced stages through the meta-analysis, in order to provide scientific reference for the design of future clinical trials.

## 2 Study aim

The aim of the systematic review and meta-analysis was to systematically evaluate the efficacy and safety of Qigong mediated therapy for the treatment of advanced PSMDs and PSSDs.

## 3 Methods

This review protocol is registered in the International Platform of Registered Systematic Review and Meta-Analysis Protocols (INPLASY). The registration number was INPLASY202007051 (DOI number is 10.37766/inplasy2020.7.0051). The systematic review will be conduct by Cochrane Handbook for Systematic Reviews of Interventions guidelines and reported according to preferred reporting items for the Systematic Review and Meta-Analysis Protocols (PRISMA-P) guidelines. The ethical approval or informed consent was not required in this study because it belongs to secondary research which based on some previously published data.

### 3.1 Dissemination plans

We will disseminate the results of this systematic review by publishing the manuscript in a peer-reviewed journal or presenting the finding at a relevant conference.

### 3.2 Eligibility criteria

#### 3.2.1 Study designs to be included

We will only include randomized controlled trials (RCTs), non-RCTs, quasi-RCTs, reviews and other types of studies will be excluded.

#### 3.2.2 Participant or population

This review includes post-stroke depression patients regardless of race, region, sex, and the phase of post-stroke and nosogenesis.

#### 3.2.3 Intervention

The main intervention are Qigong-related exercises (eg, Qigong, Baduanjin, Yijinjing, Wuqinxi). The duration and frequency of exercises are not limited.

#### 3.2.4 Comparator

There is no exclusion based on comparator method for this review, and the patients could be treated with any forms of control group including exercise, stretching, sham Qigong, waiting list control, or other treatments.

#### 3.2.5 Type of outcome

##### 3.2.5.1 Main outcome(s)

The recovery effect of Qigong training for PSMD and PSSD is evaluated by pre-post score changes of different clinical scales. The main outcome includes “Hamilton anxiety scale”, “Hamilton depression scale”, “the mental health part of the MOS item short from health survey,” “Generic Quality of Life Inventory-74,” “Center for Epidemiologic Studies Depression Scale”, and “Pittsburgh sleep quality index.”

##### 3.2.5.2 Additional outcome(s)

The additional outcomes include the effective rate and adverse events.

### 3.3 Information sources and Search strategy

Four English electronic databases including Cochrane Library, PubMed, Web of Science, Excerpt Medical Database, four Chinese electronic databases include Chinese Biomedical Literature Database, China National Knowledge Infrastructure, China Scientific Journal Database, and Wanfang and WHO International Clinical Trials Registry Platform will be systematically searched for suitable studies from their inception to 30 June 2020. Language is limited with English and Chinese. Strategy will be built according to guidance from the Cochrane handbook. Search strategy for Excerpt Medical Database is shown in Table 1, and similar strategies will be modified and applied for other electronic databases.

**Table 1:**
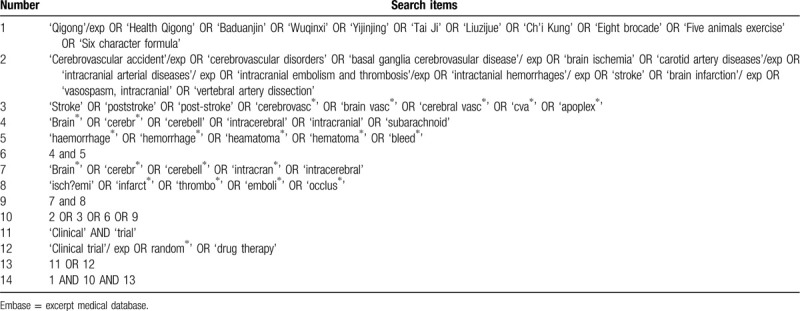
Search strategy for Embase.

### 3.4 Study selection and management

**Figure 1:**
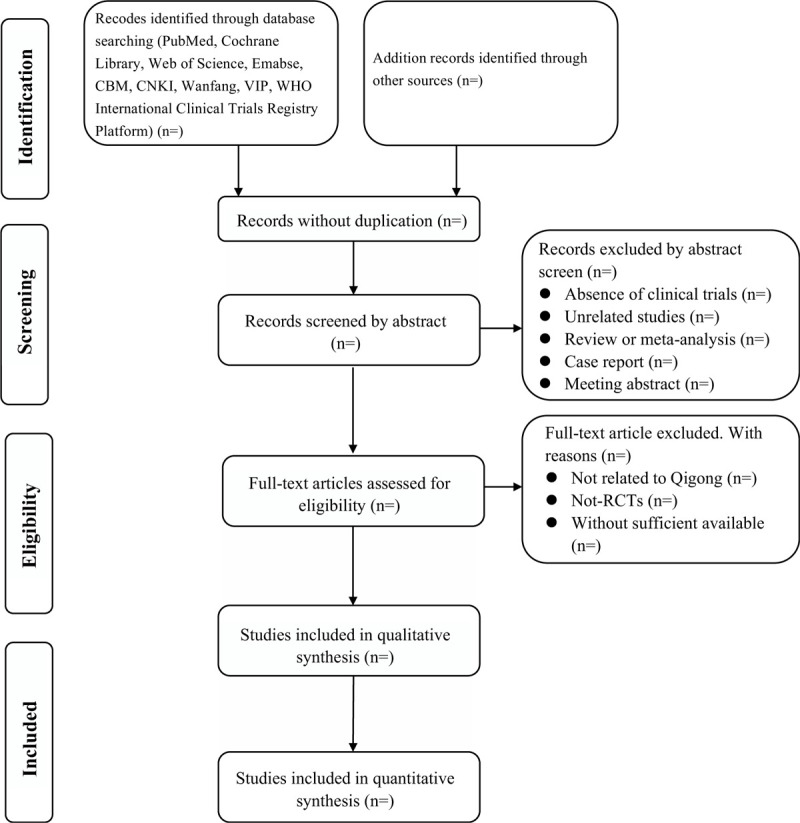
Flow diagram of literature search.

Firstly, two review authors (RZ and XD) will identify the titles and abstracts of retrieve dates independently. Endnote X9 software will be used for literature managing and records searching. Secondly, two review authors (YG and LC) will reading full text of preliminary selective articles and select suitable studies according to inclusion criteria. Finally, selecting articles will be put together. If there are some disagreements regarding inclusion and exclusion, we will make a group discussion. The details of whole selection procedure will be shown in a PRISMA flow diagram (Fig. 1).

### 3.5 Data extraction and management

Two reviewers (XD and RZ) will be charge of the data extraction according to the Cochrane Handbook for Systematic Reviews of Intervention. Information extraction form consisted mainly of following items:

(1) Publication information (the first author, year, country);
(2) Participants (sample size, source, age, gender, stroke types and severity, inclusion and exclusion criteria, and etc);
(3) Intervention (Qigong types, training frequencies and training time of every time, total training time, etc);
(4) Comparison (other forms treatments, frequencies, treatment times);
(5) Outcomes (scale instruments, assessment time, results details, adverse event, cost and funding sources).

Any disagreements will be resolved by discussion in group. The experience reviewer (YL) will make the final decision.

### 3.6 Dealing with missing data

To some extent, the missing data have an influence on study result. When we make the decision of excluding, we need to contact the authors request the missing or incomplete data that will be further check and record. If relevant data are not exit or acquired, we will exclude them from analysis.

### 3.7 Quality assessment/ risk of bias analysis

According to the guidance of the Cochrane Handbook for Systematic Review of Interventions, the quality of included RCTs will be assessed by 2 reviewers (YG and LC). The items of quality including inclusion criteria, sample size estimation, baseline, randomization, allocation sequence concealment, binding, selective reporting, missing data managements, and other bias. Evidence quality will be shown as high, unclear, low risk of bias in accordance with the criteria of the risk judgment. If there are disagreements, an experience researcher (YL) will make the decision.

### 3.8 Strategy of data synthesis

The Revman 5.3 software provided by Cochrane Collaboration is used to perform all statistical analyses. All outcomes are presented as continuous variables in this review. We will perform a pairwise meta-analysis using a random-effect model. To determine the effect size, risk rations with 95% confidence intervals will be calculated for dichotomous outcomes and the standard mean difference with 95% confidence intervals will be calculated for continuous outcomes. Depending on the heterogeneity assessed by the *I*^2^ statistic, a fixed- ore random- effect model will be used. If there is statistical heterogeneity, sensitivity to explore the source of heterogeneity.

### 3.9 Publication bias analysis

If ten or more studies are in included in the meta-analysis, we will sight publication biases and poor methodological quality by funnel plots.

### 3.10 Assessment of heterogeneity

#### 3.10.1 Sensitivity analysis

For the quality analysis, we will conduct a sensitivity analysis of main outcomes to explore an individual study's influence of bias on results.

#### 3.10.2 Subgroup and meta-regression analysis

If the heterogeneity is apparent, subgroup and meta-regression analysis will be set up according to the characteristic of the study to explore the source of heterogeneity with regard to age, gender, region, type of control interventions, type of Qigong, and frequency and duration.

## 4 Discussion

Chinese Qigong has a long history and has been widely used as an effective treatment for PMSDs treatment in China, In China and other Asian countries, people believed mind-body exercises, such as Qigong, Yoga and Meditation, are often recommended to regulate mood or emotion for stroke patients. The scientists not only focused on the survival rate of PSMD and PSSD patients, but also more pay attention to how to improve the mental quality of them. There are many systematic reviews and meta-analysis reviews reported the efficacy on stroke patients’ mood and sleep condition, including drugs, acupuncture and other treatments. To best our knowledge, there are few analyses focusing on Qigong effect on PMSDs. We hope to evaluate the efficacy of different type of Qigong on PMSDs and provide more recommendable measures for PMSDs patients. The strengths of our study include that comprehensive searching in Chinese and English database, rigorous evaluation of quality, and sensible subgroup analysis design. All of the above can make our analysis more convictive. Some limitations of this study should be noted. Our studies only search database in English and Chinese because of language barriers. So, there may exist a language bias. We will do a full-scale in the future to evaluate it better. Then, the large clinical heterogeneity may exist for different disorder stage, duration of treatment and action consistency, future study will be more noticed those limitations.

## Acknowledgments

The authors would like to thank Yuan Liu for critically reviewing the manuscript.

## Author contributions

**Data collection:** Xing Dong, Renyan Zhang

**Statistical analysis:** Ying Guo, Longfang Chen

**Supervision:** Yuan Liu

**Software:** Renyan Zhang

**Writing – original draft:** Xing Dong

**Writing – review & editing:** Yuan Liu

## Correction

When originally published, Yun Lu was included on the author list by mistake. They have since been removed.
